# Development of a multidimensional housing and environmental quality index (HEQI): application to the American Housing Survey

**DOI:** 10.1186/s12940-022-00866-8

**Published:** 2022-05-24

**Authors:** MyDzung T. Chu, Andrew Fenelon, Judith Rodriguez, Ami R. Zota, Gary Adamkiewicz

**Affiliations:** 1 Department of Environmental and Occupational Health, Milken Institute School of Public Health, The George Washington University, Washington, DC USA; 2 Institute for Clinical Research and Health Policy Studies, Tufts Medical Center, Boston, MA USA; 3 School of Public Policy and Department of Sociology and Criminology, Penn State University, University Park, PA USA; 4 Department of Architecture, Harvard University Graduate School of Design, Cambridge, MA USA; 5 Department of Environmental Health, Harvard T.H. Chan School of Public Health, Boston, MA USA

**Keywords:** U.S. housing stock, Indoor environmental exposures, Housing quality, Health equity, Development

## Abstract

**Background:**

Substandard housing conditions and hazardous indoor environmental exposures contribute to significant morbidity and mortality worldwide. Housing indices that capture the multiple dimensions of healthy housing are important for tracking conditions and identifying vulnerable households. However, most indices focus on physical deficiencies and repair costs and omit indoor environmental exposures, as few national data sources routinely collect this information.

**Methods:**

We developed a multidimensional Housing and Environmental Quality Index (HEQI) based on the World Health Organization’s Housing and Health Guidelines and applied it to the 2019 American Housing Survey (AHS). The HEQI consisted of ten domains associated with poor health: household fuel combustion, dampness and mold, pests and allergens, lead paint risk, high indoor temperatures, low indoor temperatures, household crowding, injury hazards, inadequate water and sanitation, and ventilation. We evaluated the validity and performance of the HEQI against three housing characteristics (i.e., year built, monthly rent costs, unit satisfaction rating) and two established indices (i.e., Adequacy Index, Poor Quality Index).

**Results:**

Approximately 79% (92 million) of U.S. households reported at least one HEQI domain associated with poor health (mean per household: 1.3; range: 0,8). Prevalent domains included household fuel combustion (61.4%), dampness and mold (15.9%), inadequate water and sanitation (14.3%), and injury hazards (11.9%). Pests and allergens, low indoor temperatures, and injury hazards were consistently associated with older homes, lower rent costs, and lower unit satisfaction. Compared to established housing indices, the HEQI captured four new environmental domains which enabled the identification of 57.7 million (63%) more households with environmental risk factors like mold, cockroaches, crowding, household fuel combustion, and higher building leakage.

**Conclusions:**

Indoor environmental exposures are prevalent in U.S. households and not well-captured by existing housing indices. The HEQI is a multidimensional tool that can be used to monitor indoor environmental exposures and housing quality trends in the U.S. Some domains, including radon, pesticides, asbestos, noise, and housing accessibility could not be assessed due to the lack of available data in the AHS. The mounting evidence linking residential environmental exposures with adverse health outcomes underscore the need for this data in the AHS and other national surveys.

**Supplementary Information:**

The online version contains supplementary material available at 10.1186/s12940-022-00866-8.

## Background

The World Health Organization (WHO) defines healthy housing as one that “supports a state of complete physical, mental and social well-being” [1]. Poor housing conditions and exposure to environmental hazards in the home are risk factors for adverse health, including respiratory, cardiometabolic, and reproductive health effects; physical injuries; poor mental health; and shorter life expectancy [1–4]. These housing-related health risk factors fall within the multiple dimensions of healthy housing, such as structural deficiencies (e.g. cracks in walls or ceilings, no insulation), chemical/material hazards (e.g. lead paint, asbestos, volatile organic compounds, pesticides, flame retardants, phthalates), poor indoor air quality (e.g. particulate matter, carbon monoxide, radon, environmental tobacco smoke), indoor allergens (e.g. mold, dust, pet dander), pests (e.g. rodents, cockroaches), and noise [1, 5, 6]. Exposure to these hazards are driven by the joint influence of both indoor and outdoor sources, the building design and conditions, the presence and performance of ventilation systems, and residential activity patterns, including the use of consumer products and appliances that emit environmental pollutants [7, 8]. In addition, the characteristics of the living space (e.g., thermal comfort, natural lighting, occupancy) and access to basic resources (e.g., heating, plumbing, cookstove) also contribute to residents’ health, well-being, and quality of life [1].

Environmental health indices that can capture the multiple dimensions of healthy housing are important tools for characterizing the risk of exposure as well as evaluating the effectiveness of interventions. For example, a study among low-income housing developments used a summed environmental health index to identify risk factors associated with poor self-reported health [8]. The Massachusetts Department of Public Health developed an Environmental Scoring System to evaluate changes in environmental triggers (e.g. mold, pest, smoke, dust, chemical hazards) following implementation of home-based asthma education programs [9]. In Arizona, the Phoenix Children’s Hospital conducted a visual assessment of 29 potential injury hazards and seven potential respiratory health hazards to evaluate the effectiveness of a multidisciplinary home-intervention program for low-income families [10]. Collectively, these studies underscore the need for comprehensive, evidence-based environmental health indices for housing portfolios. However, at the national level, there is a paucity of indices that capture multiple domains of housing quality and indoor environmental risk factors. In addition, data are often not routinely collected, inhibiting the ability to track housing and environmental conditions over time.

The American Housing Survey (AHS), a nationally-representative and longitudinal household survey, may provide a platform to address existing data gaps. It is the most comprehensive survey of housing conditions in the U.S. and has been widely used to track and report on housing quality, housing stability, and occupant characteristics over time. AHS variables can be mapped onto key indoor environmental exposures that influence residents’ health [7]. To date, a housing and environmental health index based on AHS data has not been developed. Established AHS indices focus solely on housing adequacy and quality, such as the Adequacy Index and the Poor Quality Index (PQI) [11, 12], and are framed around a particular way of *seeing* quality, such as the need or cost of repairs, presence of (typically major) physical deficiencies, or resident’s satisfaction with their unit [13]. These indices do not account for environmental conditions that have been associated with adverse health, such as poor indoor air quality, mold, chemical hazards, thermal discomfort, and ventilation [1].

The objective of our study was to develop a national Housing and Environmental Quality Index (HEQI) that can capture the multiple dimensions of healthy housing, particularly indoor environmental exposures. The selection of our HEQI domains was informed by the WHO Housing and Health Guidelines [1] and applied to the AHS data. We evaluated the performance of the HEQI against housing characteristics and established indices in the AHS.

## Methods

### Data

The AHS is a biennial U.S. survey of housing units since 1973 led by the U.S. Department of Housing and Urban Development (HUD). Participation in the AHS is voluntary and consists of a computer-assisted, in-person or phone interview with the main householder in primarily English and Spanish languages. Each sampled housing unit is weighted to represent approximately 50 to 15,410 housing units [14].

For our HEQI development, we used the 2019 AHS national public flat file downloaded from U.S. Census Bureau website on December 11, 2020 with a sample size of 63,185 households [15]. At the time of the analysis, the 2019 survey was the most recent data available. In Supplemental Data, we provide the variable and response coding for the HEQI using AHS data from the 2011 to 2019 survey cycles to allow for longitudinal assessment (Table S1). However, researchers should note differences in the sample design and housing units between the 2011–2013 and the 2015–2019 AHS data [16].

We excluded housing units that were vacant or if the owner had usual residence elsewhere because they were ineligible for AHS questions pertinent to our HEQI. We also excluded mobile housing types (e.g., trailers, boat, van, RV) given their low prevalence in the data (5.7% of occupied units) and to reduce heterogeneity of our sample. Our final sample consisted of 51,933 occupied housing units.

### Housing and health domain identification

We identified domains that captured indoor environmental risk factors and housing conditions associated with adverse health effects. The WHO Housing and Health Guidelines and related guidance documents recommended 15 priority domains based on systematic reviews of environmental health hazards and substandard housing conditions associated with poor health and the availability of recommendations to correct such hazards or conditions [1, 5, 6, 17–27]. The WHO guidelines provided an evidence-based health framework for our HEQI development (Table 1).

**Table 1:** World Health Organization (WHO) recommended housing and health domains

**No.**	**WHO Housing and Health Domains**	**References**	**Examples of hazards**	**Health effects**	**Data availability in the AHS**^a^
1	Indoor air quality	1,5,6,14	Particulate matter, carbon monoxide, volatile organic compounds, methane, black carbon, nitrous oxides, mold, formaldehyde	Cardiovascular, stroke, respiratory (e.g., chronic obstructive pulmonary disease, asthma, lung cancer), neurological, shortened life expectancy, poisonings, mortality	Available
2	Dampness and mold	1,15	Bacteria and fungi	Respiratory (e.g., asthma, hypersensitivity), immunologic reactions	Available
3	Pests and allergens	16	Transport of endoparasites vector and/or pathogenic organisms	Respiratory (e.g., asthma), allergies, conjunctivitis, rodent-related diseases (e.g., plague, leptospirosis, rickettsia pox, rat-bite fever, dysentery, typhus)	Available
4	Lead	1,5	Lead poisoning	Neurotoxicant, impairments to children’s cognitive and social development, elevated blood pressure, cardiovascular disease, renal insufficiency	Available
5	High indoor temperatures	1	Extreme heat stress	All-cause mortality, heat stroke, dehydration, heart and kidney co-morbidities. Older adults (age 65+) vulnerable	Limited
6	Low indoor temperatures	1,5,17	Extreme cold stress, increased risk of dampness and mold	Respiratory (e.g., chronic obstructive pulmonary disease, asthma, lung inflammation), cardiovascular (e.g., vasoconstriction, chest pain, high blood pressure), strokes, all cause-mortality, depression, subarachnoid hemorrhage	Available
7	Household crowding	1,5	Resuspension of air pollutants, infectious disease transmission, poor sanitation, noise, second-hand tobacco smoke	Poor mental health, sleep disturbance, gastroenteritis and diarrheal diseases, infectious disease, behavioral issues (e.g., hostility), domestic violence, impaired children's cognitive development	Available
8	Injury hazards	1,5,18	Fires, falls, drowning, poor storage or labelling of hazardous chemicals	Electrocutions, burns, fractures, lacerations, chemical poisonings, hospitalization, mortality	Available
9	Inadequate water and sanitation	1,19,20	Endoparasites vectors, pathogenic organisms, toxic chemicals, metals (e.g. lead, arsenic)	Cholera, diarrhea illnesses, hepatitis A, typhoid, polio, lead poisoning, dehydration	Available
10	Ventilation	1	Low building leakage traps air pollutants indoors, higher leakage increase risk for dampness, mold, pest problems, and infiltration of air pollution	*See health effects related to the WHO domains of indoor air quality, dampness and mold, pest and allergens, high and low indoor temperatures*	Limited
11	Radon^b^	1,5,21	Radon	Lung cancer, mortality	Unavailable
12	Pesticides	1,16,22	Fungicides, herbicides, biocides	Headaches, vertigo, nausea, muscular weakness, endocrine disruption, carcinogenesis, neurological and reproductive effects	Unavailable
13	Asbestos	1,23	Asbestos	Respiratory, cancer (e.g. asbestosis, mesothelioma)	Unavailable
14	Noise	1,5,24	Unwanted noise, frequency and loudness, beyond 40 dB (or 55 dB for short-term)	Cardiovascular, cognitive impairments, sleep disturbance Long-term effects: hearing damage, psychological and physiological distress, increased allostatic load	Unavailable
15	Housing accessibility for people with functional impairments^c^	1	Safety: fires, slips/trips, drowning	Electrocutions, burns, falls, chemical poisonings, burns, drowning, fractures, hospitalization, mortality	Unavailable

^a^ Data availability was based on questions asked in the national AHS 2011–2019 surveys of occupied units. ‘Available’ = The AHS asked question(s) directly relevant to this domain and asked in multiple years; ‘Limited’ = The AHS did not ask direct question(s) for this domain but the data could be combined with external sources to approximate this domain; ‘Unavailable’ = No AHS questions were asked relevant to this domain

^b^ AHS asked questions about radon in AHS subsamples

^c^ AHS asked questions about housing accessibility in specific AHS years and subsamples, and not for all five survey years, 2011–2019

### Data selection

We reviewed the availability of AHS questions (referred to as ‘AHS items’) for each of the 15 WHO domains. To ensure longevity of the HEQI, we prioritized AHS items that have been asked in multiple survey years, particularly in the most recent survey cycles (i.e., 2015–2019). AHS data was considered ‘available’ if it contained items relevant to the WHO domain for multiple survey years; ‘limited’ if items did not directly relate to the domain but proxy measures could be applied; and ‘unavailable’ if neither of these conditions were met (Table 1). From the 15 WHO domains, ten had available or limited information in the AHS (Table 2). Thus, we integrated the following ten domains into the HEQI:

1. Indoor air quality, specifically household fuel combustion
2. Dampness and mold
3. Pests and allergens, specifically rodents and cockroaches
4. Lead, specifically lead paint risk
5. High indoor temperatures
6. Low indoor temperatures
7. Household crowding, specifically severe crowding
8. Injury hazards, specifically electrical and structural integrity
9. Inadequate water and sanitation
10. Ventilation, specifically building normalized leakage

**Table 2:** Housing and environmental quality Index (HEQI): frequency distribution of AHS items for each domain and their availability in established housing indices, American Housing Survey 2019 national public file (sample *N* = 51,993)

**No**	**WHO Domains and available AHS items**	**AHS 2019 Sample distribution** N (%)	**AHS items in the Poor Quality Index (PQI)?**^**ƚ**^	**AHS items in the Adequacy Index?**^**ǂ**^
**1**	**Indoor air quality**	**32,226 (62.0%)**		
***Household fuel combustion***				
	Cooking fuel: Has piped gas or LP gas	22,137 (42.6%)	No	No
	Heating fuel: Kerosene or other liquid fuel	107 (0.2%)	Yes^**a**^	No
	Heating fuel: Coal or coke	17 (0.0%)	No	No
	Heating fuel: Wood	518 (1.0%)	No	No
	Heating type: Cooking stove to heat home	31 (0.1%)	No	No
	Heating type: Fireplace without inserts	23 (0.0%)	No	No
	Heating type: Has unvented room heaters	213 (0.4%)	No^**a**^	No
	Unit has a useable fireplace	19,137 (36.8%)	No	No
**2**	**Dampness and mold**	**8,012 (15.4%)**		
***Mold (in last 12 months)***				
	Mold in bathroom	721 (1.4%)	No	No
	Mold in bedroom	358 (0.7%)	No	No
	Mold in kitchen	248 (0.5%)	No	No
	Mold in living room	191 (0.4%)	No	No
	Mold in other room	224 (0.4%)	No	No
***Dampness (in last 12 months)***				
	Water leak from roof	2,069 (4.0%)	Yes	Yes
	Water leak from wall or closed window or door	841 (1.6%)	Yes	Yes
	Water leak from basement	1,223 (2.4%)	Yes	Yes
	Water leak with unknown inside source	155 (0.3%)	Yes	Yes
	Water leak from broken water heater	344 (0.7%)	No	Yes^**b**^
	Water leak from somewhere else outside	592 (1.1%)	Yes	Yes
	Water leak from pipes leaking	1,579 (3.0%)	Yes	Yes
	Water leak from own plumbing fixtures	934 (1.8%)	Yes	Yes
	Water leak from somewhere else inside	1,038 (2.0%)	Yes	Yes
**3**	**Pests and allergens**	**2,313 (4.4%)**		
	Evidence of rodents (daily or weekly)	836 (1.6%)	Yes^**c**^	Yes
	Evidence of cockroaches (daily or weekly)	1,713 (3.3%)	No	No
**4**	**Lead**	**805 (1.5%)**		
	Lead paint risk: Peeling paint larger than 8 × 11 inches AND year built before 1980	805 (1.5%)	Yes^**d**^	Yes^**d**^
**5**	**High indoor temperatures**	**5,482 (10.5%)**		
	No central or window air conditioning unit	5,482 (10.5%)	No	No
**6**	**Low indoor temperatures**	**2,938 (5.7%)**		
	Unit was uncomfortable cold for 24+ hours	2,938 (5.7%)	Yes	Yes
	Main heating equipment broke down 1+ times for 6 h or more	1,108 (2.1%)	Yes	Yes
**7**	**Household crowding**	**252 (0.5%)**		
	Severe crowding: Occupancy-to-room Ratio > 1.5	252 (0.5%)	No	No
**8**	**Injury hazards**	**6,014 (11.6%)**		
***Electrical***				
	No electrical wiring	19 (0.0%)	Yes	Yes
	Electrical wiring exposed	1,396 (2.7%)	Yes	No
	Not every room has working electrical plug	1,048 (2.0%)	Yes	No
	Fuse(s) blown or circuit breakers tripped 2+ times in the last 3 months	1,711 (3.3%)	Yes	Yes
***Structural Integrity***				
	Floor has holes	547 (1.1%)	Yes	Yes
	Walls or ceilings have open holes or cracks wider than dime	2,568 (4.9%)	Yes	Yes
**9**	**Inadequate water and sanitation**	**6,077 (11.7%)**		
***Water quality and quantity***				
	Unit has no hot/cold running water	120 (0.2%)	Yes	Yes
	Unit without running water in last 90 days	1,361 (2.6%)	Yes^**e**^	Yes^**e**^
	Non-public drinking water sources (e.g. individual wells)	3,874 (7.5%)	No	No
***Poor sanitation***				
	1+ toilet breakdowns within last 3 months that lasted 6 h or more	659 (1.3%)	Yes	Yes
	1+ sewer breakdowns within last 3 months that last 6 h or more	497 (1.0%)	Yes	No
	Unit has NO bathtub OR shower OR no flush toilet	48 (0.1%)	Yes	Yes
	Unit does NOT have working kitchen sink	92 (0.2%)	Yes^**f**^	No
**10**	**Ventilation**	**1,624 (3.1%)**		
	High building leakage^**c^**^: Normalized leakage > 2.5	1,624 (3.1%)	No	No

^Ɨ^ Eggers, F. J., & Moumen, F. (2013a). American Housing Survey: A Measure of (Poor) Housing Quality. US Department of Housing and Urban Development. Access at: www.census.gov/programs-surveys/ahs/research/publications/PoorHousingQuality.html

^ǂ^ Eggers, F. and Moumen, F. (2013b). American Housing Survey: Housing adequacy and quality as measured by the AHS. Available at SSRN 2,284,174. 2013 Mar 1. Access at: https://www.census.gov/content/dam/Census/programs-surveys/ahs/publications/HousingAdequacy.pdf

^^^Development adapted from Chan, W. R., Joh, J., & Sherman, M. H. (2013). Analysis of air leakage measurements of US houses. Energy and Buildings, 66, 616–625

^a^ PQI only asked about 'Main heating equipment as unvented kerosene heater(s)'

^b^ Unclear which inside and outside water leak questions were included. For comparison, we assumed that all AHS items related to water leaks were included

^c^ Item was grouped with the component 'Inside structural or other problems'

^d^ The only AHS item used was whether unit has an area of peeling paint larger than 8 x 11 inches, irrespective of year built

^e^ Item was not asked of units with no hot/cold running water. The PQI counts each time the unit “is completely without water"

^f^ Item was grouped with the component ‘Kitchen Problems’

The final HEQI consisted of 43 AHS items across these ten domains. The four HEQI domains of dampness and mold, low indoor temperatures, household crowding, and inadequate water and sanitation were well-captured by the AHS based on the relevance and robustness of items included (Table 2). For the remaining six HEQI domains, household fuel combustion, pest and allergens, lead paint risk, high indoor temperatures, injury hazards, and ventilation, the relevance and precision of AHS items to approximate the underlying domain-specific hazard varied. The AHS does not ask specifically about combustion activities, household crowding, lead exposure, periods of high indoor temperature, or ventilation factors such as insulation or building leakage. Therefore, we had to approximate these domains using available AHS data or external data sources.

For the household fuel combustion, we used the presence of cooking and heating appliances with specific fuel types (e.g. gas, wood, kerosene) as surrogates for actual use. Many studies have found elevated indoor air pollutant concentrations from the presence of gas stoves and fireplaces [28–32]. Although gas-fueled home and water heaters also emit significant combustion pollutants [31, 33], we did not include them in the HEQI because they are required to be ventilated and are often located away from occupants’ main living space [34, 35]. We also created a severe crowding indicator that identified households with 1.5 or more persons per room, consistent with the U.S. Census and HUD’s definition of severe crowding [36, 37]. We also created an indicator for lead paint risk to identify housing units built before 1980 that also had peeling paint larger than 8 × 11 inches. We selected the year threshold of 1980 to temporally align with the ban of lead from residential paint starting in 1978 [38]. High indoor temperatures was flagged if the household reported not having central air or window air conditioning unit(s), which are mechanical controls known to reduce the risk of health-related illnesses [39, 40]. Ownership of these controls could vary across geographic regions and climate types [41] and thus, we controlled for U.S. Census regions in regression analyses.

Lastly, the ventilation domain was based on an approximation of building leakage (i.e., normalized leakage), which is a measure of building envelope airtightness relative to its size and height [42]. To create the building leakage indicator, we adapted methods by Chan et al. (2013) and accounted for year built, unit size and height, basement and foundation types, and the International Energy Conservation Code (IECC) Climate Zones [42]. Data on weatherization assistance program, basement foundation type, and IECC Climate Zones were not available in the AHS and had to be approximated based on income-to-poverty thresholds, county-level IECC estimates, and the 2009 Residential Energy Consumption Survey, respectively. See Supplemental Data Appendix 1 for more details about the building leakage indicator.

We excluded several AHS items from the HEQI. Mold in the basement and outside infrastructural problems were only asked of single-family households and thus excluded. For domains with multiple AHS items, items that were moderately or strongly correlated (Spearman *r* >|0.60|) were removed to reduce collinearity. Specifically, we selected wood heating fuel over wood heating type (*r* = 0.69) [6] and drinking water from a non-public source over septic tank or cesspool sewer system (*r* = 0.63) [43, 44] because they were more inclusive and better approximated the underlying hazard. The HEQI AHS item codebook can be found in Supplemental Table S1. Inter-item correlations within each HEQI domain and inter-domain correlations can be found in Supplemental Tables S2.

### Data reduction

All 43 AHS item responses were binary coded (yes/no) to indicate prevalence of the HEQI risk factor. Within each HEQI domain, AHS item responses were summed to create a domain-specific score. Each AHS item contributed equally to the domain score (i.e. equal weights). For each HEQI domain, we then created a binary indicator to flag the presence of one or more underlying risk factor. The practical consideration for our approach was to allow for estimation of the population prevalence for each HEQI domain.

We also created a cumulative HEQI score by summing HEQI domains with at least one risk factor present (range 0–10). While we considered health-based weighting schemes for the HEQI, we chose an equal weights approach. Each domain represented a unique set of hazards (e.g. physical, chemical, biological) with diverse health effects, as shown in Table 1, and thus was treated as equally important for our multidimensional index. As such, the cumulative HEQI score reflected the types of residential health hazards present instead of the degree of health severity. A score of 0 indicated that none of the domain-specific hazards were reported while a score of 10 indicated that ten different domain-specific hazards were reported in the AHS. This index can thus be used to inform and prioritize residential interventions to improve health across U.S. housing portfolios.

Generating weights that account for the health severity of each AHS item was empirically and methodologically challenging and required estimation of the average causal effect for each indicator/domain based on the same outcome, time period, and representative sample [45]. However, the AHS does not routinely collect data on health outcomes. Furthermore, the epidemiological evidence for each indicator and domain varied by study designs, sample populations, and target outcomes [1, 12]. These factors limited our ability to estimate and compare the relative degree of health severity across indicators and domains.

### Validity testing

We assessed the validity of the HEQI using several approaches. We computed polychoric  correlation matrices to evaluate the intra- and inter-domain correlations and the extent to which each domain captured a unique latent feature of healthy housing (i.e. *discriminant validity*) (Table S2) [46]. Secondly, we evaluated *criterion validity*, specifically *predictive validity*, of the HEQI and its association with housing characteristics like unit rating, year built, and monthly rent costs, consistent with previous studies [13, 47, 48]. The AHS item on unit rating asked: “On a scale of 1 to 10, how would you rate your unit as a place to live?”, with 10 ranked as the best place to live. Year built was categorized as intervals (< 1960 (ref), 1960–1969; 1970–1979; 1980–1989; 1990–1999; 2000 +). We ran ordinal logistic regressions to examine the association of the ten HEQI domains (independent variables) with unit rating and year built (dependent variables) separately. For the analysis with year built, we excluded the lead paint risk domain to avoid endogeneity with the outcome. Monthly rent costs (dollar units) was continuous, had a right-tailed distribution, and log-transformed prior to modeling. We used linear regression to assess the association of HEQI domains and monthly rent costs among renter households (*N* = 20,205).

Third, we evaluated *convergent validity*, i.e. the extent to which our index aligned with the two established AHS housing indices: Adequacy Index and Poor Quality Index (PQI). The Adequacy Index (named ZADEQ prior to the 2015 AHS) is comprised of eight criterion capturing severe physical deficiencies like no running water, plumbing facilities, heating, or electricity, and signs of structural weaknesses like water leaks and presence of rats [11]. Units are categorized as ‘adequate’, ‘moderately inadequate’, or ‘severely inadequate’ based on the prevalence of these risk factors and their combinations. The PQI is comprised of 35 AHS items that fall into eight housing quality domains, such as electricity, heating, inside and outside structural hazards, bathroom, kitchen, water and sewer, and elevator problems [12]. The PQI items for ‘No working elevator in building of 4 + stories’ and outside infrastructural problems were excluded from our comparisons because they were not available in recent AHS survey cycles and not asked of multifamily households, respectively. Although the PQI assigned differential weights to each AHS item based on severity of the risk factor, we used an equal weighting approach for all comparisons. Across the three indices, we compared the number of U.S. households with at least one risk factor in each HEQI domain (i.e., HEQI count ≥ 1, PQI count ≥ 1, and Adequacy categories of ‘moderately’ or ‘severely’ inadequate).

### Statistical analyses

We ran descriptive statistics summarizing the frequency and means of the cumulative and domain-specific HEQI scores. We also estimated the prevalence of HEQI domains by housing characteristics that may modify indoor air pollution levels, such as multifamily status and tenant-based/non-homeownership households (Table 3) [2, 7]. We also stratified by unit square footage and normalized leakage values below 1.0, an indicator of building airtightness (Table S3), and by whether the households had children under the age of 18, a vulnerable population susceptible to adverse health effects from residential hazards (Table S4), in order to better approximate the risk burden in the U.S. population. To obtain weighted estimates of the U.S. population and associated standard errors, we applied the WEIGHT variable and 160 replicate weights using the balanced repeated replications (BRR) method [49] in the R *survey* package [50].

**Table 3:** Distribution of U.S. households (in the thousands) with at least one HEQI risk factor in each domain by housing tenure and building type, American Housing Survey 2019 national public file (sample *N* = 51,993)

	**All Households**	**Homeowner**	**Non-Homeowner**^a^	**Single-family**	**Multifamily**
	(*N* = 117,284)	(*N* = 74,337)	(*N* = 42,947)	(*N* = 85,817)	(*N* = 31,467)
**Cumulative**					
Count ≥ 1, weighted N (%)	92,043 (78.5%)	62,336 (83.9%)	29,707 (69.2%)	71,221 (83%)	20,822 (66.2%)
1 Domain	53,483,340 (45.6%)	37,068 (49.9%)	16,415 (38.2%)	41,647 (48.5%)	11,836 (37.6%)
2 Domains	26,119,877 (22.3%)	18,052 (24.3%)	8,068 (18.8%)	20,527 (23.9%)	5,593 (17.8%)
3 Domains	8,482,142 (7.2%)	5,299 (7.1%)	3,183 (7.4%)	6,384 (7.4%)	2,099 (6.7%)
4-8 Domains	3,958,041 (3.4%)	1,917 (2.6%)	2,041 (4.8%)	2,664 (3.1%)	1,294 (4.1%)
Mean (Min, Max)	1.27 (0, 8)	1.31 (0, 8)	1.20 (0, 8)	1.32 (0, 8)	1.12 (0, 8)
**Household fuel combustion**					
Count ≥ 1, weighted N (%)	71,962 (61.4%)	53,189 (71.6%)	18,773 (43.7%)	59,558 (69.4%)	12,404 (39.4%)
Mean (Min, Max)	0.81 (0, 4.0)	1.0 (0, 4.0)	0.51 (0, 4.0)	0.98 (0, 4.0)	0.43 (0, 3.0)
**Dampness and mold**^b^					
Count ≥ 1, weighted N (%)	18,699 (15.9%)	11,251 (15.1%)	7,449 (17.3%)	13,743(16%)	4,956 (15.8%)
Mean (Min, Max)	0.20 (0, 9.0)	0.18 (0, 8.0)	0.24 (0, 9.0)	0.20 (0, 8.0)	0.22 (0, 9.0)
**Pests and allergens**					
Count ≥ 1, weighted N (%)	4,949 (4.2%)	1,759 (2.4%)	3,190 (7.4%)	2,703 (3.1%)	2,246 (7.1%)
Mean (Min, Max)	0.049 (0, 2.0)	0.024 (0, 2.0)	0.087 (0, 2.0)	0.034 (0, 2.0)	0.084 (0, 2.0)
**Lead paint risk**					
Count = 1, weighted N (%)	1,952 (1.7%)	939 (1.3%)	1,013 (2.4%)	1,329 (1.5%)	623 (2.0%)
Mean (Min, Max)	0.015 (0, 1.0)	0.012 (0, 1.0)	0.021 (0, 1.0)	0.014 (0, 1.0)	0.019 (0, 1.0)
**High indoor temperatures**					
Count = 1, weighted N (%)	10,344 (8.8%)	5,343 (7.2%)	5,001 (11.6%)	6,435 (7.5%)	3,909 (12.4%)
Mean (Min, Max)	0.11 (0, 1.0)	0.082 (0, 1.0)	0.14 (0, 1.0)	0.085 (0, 1.0)	0.15 (0, 1.0)
**Low indoor temperatures**					
Count ≥ 1, weighted N (%)	6,841 (5.8%)	3,745 (5.0%)	3,097 (7.2%)	4,857 (5.7%)	1,984 (6.3%)
Mean (Min, Max)	0.078 (0, 2.0)	0.065 (0, 2.0)	0.097 (0, 2.0)	0.073 (0, 2.0)	0.088 (0, 2.0)
**Severe crowding**					
Count = 1, weighted N (%)	507 (0.4%)	117 (0.2%)	390 (0.9%)	223 (0.3%)	284 (0.9%)
Mean (Min, Max)	0.0048 (0, 1.0)	0.0018 (0, 1.0)	0.0093 (0, 1.0)	0.0027 (0, 1.0)	0.0096 (0, 1.0)
**Injury hazards**					
Count ≥ 1, weighted N (%)	13,959 (11.9%)	7,873 (10.6%)	6,087 (14.2%)	9,897 (11.5%)	4,063 (12.9%)
Mean (Min, Max)	0.14 (0, 5.0)	0.12 (0, 5.0)	0.17 (0, 5.0)	0.13 (0, 5.0)	0.16 (0, 5.0)
**Inadequate water and sanitation**					
Count ≥ 1, weighted N (%)	16,802 (14.3%)	12,561 (16.9%)	4,241 (9.9%)	14,303 (16.7%)	2,499 (7.9%)
Mean (Min, Max)	0.13 (0, 4.0)	0.14 (0, 4.0)	0.10 (0, 4.0)	0.14 (0, 4.0)	0.090 (0, 4.0)
**High building leakage**					
Count = 1, weighted N (%)	2,813 (2.4%)	596 (0.8%)	2,218 (5.2%)	536,183 (0.6%)	2,277 (7.2%)
Mean (Min, Max)	0.031 (0, 1.0)	0.010 (0, 1.0)	0.063 (0, 1.0)	0.0061 (0, 1.0)	0.088 (0, 1.0)

Weighted estimates are per 1,000 households, rounded to nearest thousandth

^a^ The non-homeowner group includes renters (96.9%) and those occupied without payment of rent (3.1%)

^b^ Missing nine respondents

All regression models were adjusted for building type (single-family [ref] vs. multifamily); housing tenure (homeowner [ref] vs. renter/live without pay); race/ethnicity of the main householder (white non-Hispanic [ref], Asian non-Hispanic, Black non-Hispanic, and Latina/o/Hispanic); educational attainment of the main householder (Bachelor/Graduate degree [ref], high school graduate/some college experience, and less than high school), and U.S. Census regions (Pacific [ref], East North Central, Middle Atlantic, New England, Middle Atlantic, South Atlantic and East South Central; and West Central). Statistical significance was set at a two-sided alpha-level of 0.05. All analyses were conducted in *R* (R Core Team, Vienna, Austria).

## Results

Based on the 2019 national AHS, approximately 78.5% (92.0 million) of U.S. households reported at least one HEQI domain and approximately 10.6% (12.4 million) reported at least three HEQI domains. Prevalent HEQI domains were household fuel combustion (61%), dampness and mold (16%), inadequate water and sanitation (14%), and injury hazards (12%) (Table 3). Major individual risk factors reported were prevalence of gas cookstoves, a working fireplace, non-public drinking water sources, and the unit being uncomfortably cold for 24 or more hours (Table 2). HEQI domains with the lowest prevalence were lead paint risk (1.7%) and severe crowding (0.4%) (Table 3).

We also assessed the burden of risk across housing types and household characteristics. Homeowner (72%) and single-family (69%) households reported a higher prevalence of household fuel combustion sources compared to non-homeowner (44%) and multifamily (39%) households, respectively. In contrast, non-homeowner and multifamily households reported a higher prevalence of high indoor temperatures (12% vs. 7%), pest and allergens (7% vs. 2–3%), and high building leakage (5–7% vs. 1.0%) (Table 3). By unit size and normalized leakage (NL) thresholds, approximately 5.9 million households lived in small, air tight units (< 1.0 NL and < 1,000 square feet). Thirty-five percent of these households reported at least one household fuel combustion source, 13% reported high indoor temperatures, and 12% reported dampness or mold hazards (Table S3). Among the 34 million households with children under the age of 18, the most common HEQI hazards reported were household fuel combustion (65%), dampness and mold (19%), injury hazards (14%), and inadequate water and sanitation (14%) (Table S4).

We found that the HEQI had good discriminant validity to capture unique dimensions of healthy housing as indicated by the low correlation coefficients across the ten HEQI domains (range: -0.05 to 0.17) (Table S2). Household fuel combustion was slightly negatively correlated with high building leakage (-0.10), while dampness and mold was slightly positively correlated with several domains: pest and allergens (0.13), low indoor temperatures (0.16), lead paint risk (0.16), and injury hazard (0.17) (Table S2).

We also found good criterion validity of HEQI domains with AHS characteristics of unit rating, year built, and monthly rent costs. Most HEQI domains were negatively associated with higher unit rating and newer buildings (Table 4): specifically, pests and allergens, lead paint risk, and injury hazards with higher unit rating (adjusted odds ratios [aORs] ≤ 0.66], and high indoor temperatures, high building leakage, and pest and allergens with newer buildings (aORs ≤ 0.70). Most HEQI domains had modest associations with monthly rent costs. The exceptions were high building leakage, which was negatively associated with monthly rent costs (adjusted relative ratio [aRR]: 0.77 (95% CI: 0.73, 0.80) and severe crowding, which was positively associated (aRR: 1.26, 95% CI: 1.13, 1.42). In addition, the direction of associations for the HEQI domains were generally consistent across the three housing characteristics. Household fuel combustion was positively associated with higher unit rating, newer housing, and higher monthly costs (aORs and aRR: 1.03–1.09), while pests and allergens (0.46–0.96), low indoor temperatures (0.80–0.94), and injury hazards (0.66–0.97) were negatively associated with these characteristics (Table 4).

**Table 4:** Associations between HEQI domain scores and AHS housing characteristics, American Housing Survey 2019 PUF (sample *N* = 51,993)

	**Rating of unit as a place to live**	**Year built**	**Monthly rent costs**
	*Ordinal scale: 1: Worse place to live, 10: Best place to live*	*Ordinal categories: *< *1960 (ref), 1960–1969 1970–1979, 1980–1989 1990–1999, 2000 and later*	*U.S. Dollars*
	Odds Ratio (95% CI)	Odds Ratio (95% CI)	Relative ratio^a^ (95% CI)
Household fuel combustion	**1.09 (1.06, 1.12)**	**1.03 (1.01, 1.06)**	**1.09 (1.06, 1.11)**
Dampness and mold	**0.68 (0.66, 0.70)**	**0.81 (0.79, 0.84)**	1.01 (0.99, 1.02)
Pests and allergens	**0.46 (0.42, 0.49)**	**0.70 (0.65, 0.75)**	**0.96 (0.92, 0.99)**
Lead paint risk	**0.50 (0.43, 0.57)**	^b^NA	0.93 (0.86, 1.01)
High indoor temperature	**0.74 (0.70, 0.78)**	**0.45 (0.42, 0.47)**	1.00 (0.97, 1.03)
Low indoor temperature	**0.80 (0.76, 0.84)**	**0.80 (0.77, 0.84)**	**0.94 (0.91, 0.97)**
Severe crowding	**0.70 (0.55, 0.89)**	0.89 (0.70, 1.11)	**1.26 (1.13, 1.42)**
Injury hazards	**0.66 (0.64, 0.69)**	**0.89 (0.85, 0.92)**	**0.97 (0.95, 0.99**)
Inadequate water and sanitation	**1.06 (1.01, 1.11)**	**1.16 (1.11, 1.21)**	1.00 (0.96, 1.03)
High building leakage	**1.27 (1.15, 1.41)**	**0.26 (0.23, 0.28)**	**0.77 (0.73, 0.80)**

^a^ Estimates represent the mean change in monthly rent costs per 1-count increase in a HEQI risk factor

^b^ Data not shown since the lead paint risk domain includes year of construction

**Bolded** are statistically significant associations (*p* < 0.05)

Models adjusted for all HEQI domains simultaneously to account for their potential correlations, as well as householder race/ethnicity, education, number of rooms in unit, multifamily status, U.S. Census divisions, and survey year

Compared to the PQI and Adequacy Index, the HEQI identified at least 57.7 million more U.S. households with one or more residential health hazards. Specifically, out of the 43 AHS items in the HEQI, the PQI overlapped with 25 items (58%) and the Adequacy Index overlapped with 21 items (49%) (Table 2). The PQI and Adequacy Index did not capture the three HEQI domains of severe crowding, high building leakage, and high indoor temperatures which impacted approximately 13 million U.S. households. Additionally, the Adequacy Index did not capture information about household fuel combustion sources, while the PQI only captured the AHS item for ‘kerosene heating’. This resulted in an underestimation of at least 71.7 million households with potential household fuel combustion sources (Table 5). For the remaining domains, the Adequacy Index and PQI varied in their capture of specific risk factors. For example, they did not include items about mold, which underestimated approximately 1.7 million households compared to the HEQI. For the pest and allergens domain, they did not ask about cockroaches, which underestimated approximately 3 million households. For the domains of low indoor temperatures, injury hazards, and water and sanitation, the PQI captured almost all of the same underlying risk as the HEQI (86–100% overlap) (Table 2).

**Table 5:** Distribution of U.S. households (in the thousands) with at least one risk factor identified by the Housing and Environmental Quality Index (HEQI), Poor Quality Index (PQI), and Adequacy Index across domains, American Housing Survey 2019 PUF (sample *N* = 51,993)

	**HEQI**	**PQI**	**Difference**		**Adequacy Index**	**Difference**	
			*PQI—HEQI*			*Adequacy Index—HEQI*	
	Total (SE)^a^	Total (SE)^a^	Count^a^	Percent	Total (SE)^a^	Count^a^	Percent
Cumulative	92,043 (184)	34,314 (142)	-57,730	-63%	30,646 (143)	-61,397	-67%
Household fuel combustion	71,962 (195)	311 (17)	-71,651	-99%	NA	-71,962	-99%
Dampness and mold	18,699 (129)	16,955 (123)	-1,745	-9.0%	16,954 (123)	-1,745	-9.0%
Pests and allergens	4,949 (70)	1,946 (45)	-3,001	-61%	1,946 (45)	-3,003	-61%
Lead paint risk^b^	1,952 (48)	2,423 (54) ^b^	471	+ 19%	2,423 (54) ^b^	471	+ 19%
High indoor temperature	10,344 (140)	NA	-10,344	-100%	NA	-10,344	100%
Low indoor temperature	6,841 (76)	6,841 (76)	0	0%	6,841 (76)	0	0%
Severe overcrowding	507 (19)	NA	-507	-100%	NA	-507	-100%
Injury hazards	13,959 (127)	13,959 (127)	0	0%	10,073 (98)	-3,887	-28%
Inadequate water and sanitation	16,802 (160)	5,455 (54)	-11,346	-68%	4,525 (55)	-12,277	-73%
High building leakage	2,813 (43)	NA	-2,813	-100%	NA	-2,813	-100%

^a^ Weighted estimates are per 1,000 households, rounded to nearest thousandth

^b^ The PQI and Adequacy Index did not have a lead paint risk domain. They did include the AHS item of ‘Peeling paint larger than 8 × 11 inches’, which we used to calculate weighted estimates and estimate differences with the HEQI

## Discussion

Substandard housing conditions and hazardous indoor environmental exposures contribute to significant morbidity and mortality worldwide [1]. Despite their known adverse health effects, most national surveys and housing indices do not collect this information. Our study developed a national, multidimensional Housing and Environmental Quality Index (HEQI) informed by the WHO’s Housing and Health Guidelines and composed of ten domains addressing structural deficiencies, indoor environmental exposures, and building conditions associated with adverse health. Using the 2019 AHS data, the HEQI identified approximately 92 million (79%) U.S. households with one or more HEQI risk factors. Compared to established housing indices, the HEQI captured four new environmental health domains of household fuel combustion, high indoor temperatures, severe crowding, and high building leakage, which enabled the identification of 57.7 million (63%) more households at risk.

The multidimensional HEQI performed better than established housing indices at capturing both housing quality and environment health risk factors. Established indices focus primarily on physical deficiencies, costs of repair, or the deflation in home values as a result of these deficiencies [11–13, 47, 48]. In particular, the PQI and Adequacy Index failed to capture environmental risk factors like mold, cockroaches, household crowding, household fuel combustion, and higher building leakage. Moreover, although prior studies have used the AHS to characterize environmental risk factors, most have focused on single AHS items like thermal comfort [51], air exchange [52], wood combustion [53], mold [54], and pests [55]. To our knowledge, the HEQI is the first multidimensional index that captures a range of housing quality and environmental health risk factors.

Prevalent environmental risk factors identified by the HEQI and not well-captured by established housing indices were household fuel combustion and dampness and mold. Household fuel combustion sources include gas cookstoves, wood and kerosene heating fuel, and fireplaces. These sources emit carbon monoxide, particulate matter, nitrogen dioxide, and other hazardous air pollutants that are associated with adverse cardio-respiratory health effects, particularly among children and the immunocompromised [56–58]. Approximately 72 million (61.4%) U.S. households reported the presence of least one household fuel combustion source, particularly gas cookstoves and fireplaces. Due to limitations of the AHS data, we did not have information about modifiers of emission levels such as appliance type, frequency of appliance use, and the presence and use of ventilation controls in order to better quantify the level and duration of exposure. However, we did observe that homeowners, single-family households, and households with children reported higher prevalence of household fuel combustion sources and lived in more airtight buildings, suggesting that these households may have a higher risk of exposure and could be prioritized for intervention efforts. Even so, the high prevalence of fuel combustion sources across *all* U.S. households emphasizes the need for more questions about indoor air quality in the AHS and other national surveys to more accurately quantify residential exposure.

Mold and damp environments were reported by approximately 18.7 million (15.9%) households, particularly water leaks from the roof, basement, and pipes, and mold in bathrooms. Mold spores can enter the indoor environment through building openings (e.g., doorways, windows, cracks, HVAC systems) and thrive in damp areas with excessive moisture, leaks, and flooding events [18, 54]. Mold triggers allergic symptoms, eczema, respiratory infections, asthma, dyspnea, and other pulmonary diseases [18, 59, 60]. Given that U.S. households spend approximately 87% of their time indoors [61], the risk of chronic exposure to these residential hazards are high, and particularly in the wintertime when the building envelope is more sealed [62].

Our study found that the HEQI had good discriminant and criterion validity to capture unique dimensions of housing and environmental quality. The inverse correlation of household fuel combustion with the building leakage domain reflects known trade-offs in indoor air quality and energy efficiency. In homes with frequent combustion-source activities (e.g., smoking, cooking, or candle/incense use) and without proper ventilation controls, building airtightness can trap air pollutants resulting in higher indoor concentrations. At the same time, high building leakage increases the risk of dampness, mold, pest problems, and energy loss [63–66]. Structural deficiencies can also lead to moisture, mold, and physical injuries; energy loss resulting in lower indoor temperatures; and openings for pests. As such, we observed positive correlations between domains affected by building structural integrity, such as dampness and mold, low indoor temperatures, lead paint risk, and injury hazards. Surprisingly, dampness and mold was not correlated with building leakage. This may be due to spatial imprecision of the building leakage indicator, which was based on regional U.S. estimates, or its coarseness as a binary indicator.

Furthermore, the HEQI was associated with household characteristics such as unit rating, year built, and rent costs. Unit rating is a consumer rating index capturing residents’ perception of well-being and quality of life [67]. In our study, risk factors strongly associated with lower unit satisfaction generally included those that residents were able to directly observe or experience, such as pests and allergens, lead paint risk, and injury hazards, consistent with a previous study [13]. Older housing is a known risk factor for physical deficiencies and chemical hazards [68]. While we could not evaluate chemical hazards, we found that older housing was strongly associated with physical deficiencies like inadequate water and sanitation, higher building leakage, and high indoor temperatures attributed to no central air or window air conditioning units. Rent costs is a market value index that assigns a monetary value to the quality of housing and neighborhood amenities, with higher rents suggestive of better quality [48]. In our study, the modest associations between HEQI domains and monthly rent costs could be due to the omission of neighborhood amenities from our analyses [48]. However we still found significant negative associations with rent costs for four HEQI domains of pests and allergens, low indoor temperatures, injury hazards, and high building leakage. Additionally, severe crowding was significantly associated with higher rent costs, consistent with previous findings that cost-burdened residents doubled-up to save on rent [2, 69, 70].

Our study also yielded findings that inform areas for future research. Household fuel combustion and high building leakage were positively associated with higher unit satisfaction. Since we did not have direct measures of indoor air quality or building leakage, we used proxy measures such as cooking and heating appliances and building features (e.g., unit size and height, basement and foundation type, year built). In effect, the positive associations with unit satisfaction may reflect residents’ preferences instead of (or despite) an understanding of the potential health risks. Indoor air pollution levels and building ventilation are generally difficult to observe without the assistance of sensor technology [71]. In addition, both of these domains have not traditionally been included in housing quality indices. Our findings underscore the need for further education among residents and housing practitioners about the sources of and strategies for reducing indoor air pollution and improving building ventilation.

We also found positive associations of inadequate water and sanitation with unit satisfaction and year built, which was primarily driven by the high proportion of households with non-public drinking water sources such as individual wells (7.5%) (exclusion of this item switched the coefficient direction of this domain to be negative, data not shown). Non-public drinking water sources are not regulated by the U.S. Environmental Protection Agency under the Safe Drinking Water Act [72] and have been associated with a higher risk of waterborne illnesses [43, 44, 73]. The positive association between non-public water sources and higher unit satisfaction may be attributed to suburban housing status, since suburbs that have a higher percentage of newer construction [74]. Unfortunately, we were not able to investigate this given the lack of information about urban/suburban status in the public AHS data in recent survey cycles.

Next, we evaluate the utility of the AHS to capture healthy housing domains recommended by the WHO and make recommendations for areas of improvement. Five domains were not captured by the AHS due to the lack of data across survey years: radon, pesticides, asbestos, noise, and housing accessibility. These housing and environmental risk factors have been widely associated with adverse health effects (Table 1) [1] and should be ascertained in future national surveys. The four HEQI domains of dampness and mold, low indoor temperatures, household crowding, and inadequate water and sanitation were well-captured by the AHS and should be continued in future surveys to allow for longitudinal assessments. The remaining six HEQI domains, household fuel combustion, lead paint risk, pest and allergens, high indoor temperatures, injury hazards, and ventilation, were roughly approximated and likely imprecise. These domains could be improved with more questions added in future surveys.

In particular, the household fuel combustion domain could be improved with more direct questions about the frequency and intensity of source activities such as cooking, heating, smoking, and candle and incense use that can contribute to higher indoor air pollution concentrations [6, 17]. Questions about appliance efficiency, furniture and flooring types, kitchen size, and the types of ventilation controls like kitchen and bathroom exhaust fans are also important determinants of indoor air quality. Despite the potential imprecision of our household fuel combustion measure, studies have found an independent association between the presence of gas stoves and higher nitrogen dioxide concentrations [28–31]. In addition, the majority of U.S. households have access to piped or bottled natural gas fuel, with the number of natural gas consumers increasing since the 1980s [75]. As such, the risk of exposure to gas combustion by-products, particularly from cooking, could be even higher. Based on the 2019 AHS, approximately 70% of U.S. households consumed natural gas, with 57% of these households using it for cooking [76]. More homes may opt for gas cooking appliances in the future. In addition, there is no federal requirement for mechanical kitchen ventilation in residential spaces [31], and even in homes with stove exhaust vents or range hoods, the quality and the degree of use during cooking events are highly variable [77–79]. Similarly, an experimental evaluation of an enclosed wood fireplace still found elevated particulate concentrations emitted into the living space [32]. Therefore, the use of a surrogate measure based on cooking and heating fuel and appliance type provides a baseline estimate of potential households at risk for indoor air pollution exposure.

In addition, our estimate of lead-paint risk at 1.7% (1.95 million) of U.S. households is likely a conservative estimate of the proportion of households with lead-based paint hazards. We used a two-fold criteria based on whether the home was built prior to 1980 and the presence of peeling paint size 8 × 11-inches or larger. This latter criteria is likely too stringent because lead-based paint can peel and crack at smaller sizes and crumble into dust [38]. To better capture lead-paint risk, future surveys should consider adding response options for smaller surface areas of peeling/cracked paint or dust. In addition, our estimate likely underestimates the risk of lead exposure overall given the lack of AHS data on other residential sources of lead, such as in soil, dust, and drinking water. Prior field-based studies using residential dust wipe samples, paint measurements, and soil samples to measure lead-based paint hazards in U.S. housing found a much higher prevalence of at-risk households: 35% (38 million) in 2000 [80] and 22% (23.2 million) in 2005–2006 [81]. The later study also found that 93% of homes with lead-based paint were built before 1978 [81]. In our study, 53% of occupied housing units were built before 1980. Based on these findings, it possible that the more accurate estimate of U.S. households with lead-based paint risk may be between 1.7% and 53%. Our lower estimate could also reflect the turnover in old housing stock nationally from renovations and newer construction [74, 82], which may have also contributed to decreasing blood lead concentrations in the U.S. population over time [83].

For the pests and allergens domain, the AHS only asked about the presence and frequency of rodents and cockroaches. Future surveys should consider other pests and allergens such as bed bugs and pet dander [19]. For the injury hazards domain, only information about electrical hazards and building integrity were collected in the AHS across multiple survey years. Information about smoke and carbon monoxide detectors, stairs and window railings, pool safety, and chemical storage were asked in previous AHS cycles but discontinued in recent years or only asked among a sub-sample of households. Going forward, these questions should be asked on a routine basis and among all households. The domain for high indoor temperatures was inferred from AHS items on central air and window air conditioning. High indoor temperatures could also be influenced by ambient temperatures and humidity, which could not be ascertained in the AHS [41, 84]. Future surveys should include direct questions about heat stress (e.g., unit was uncomfortably hot for 24 + hours) and usual temperature in the home. The ventilation domain should also include more questions about the types and performance of natural and mechanical ventilation controls (e.g., bathroom and kitchen exhausts, number of doors and windows, frequency of window opening). In addition, information about climate conditions, basement foundation type, and weatherization are needed to more accurately estimate building leakage. Lastly, routine data collection about energy efficiency (e.g., insulation, solar panels, Energy Star ratings) is important to track cost-savings and understand adaptation strategies to address climate change.

The AHS data is also subject to limitations common to national surveys. AHS items were self-reported and may be susceptible to recall error or social desirability bias. The AHS survey design is based on a federally-sponsored in-person and telephone survey, which may underestimate households in precarious or temporary housing arrangements. These issues could impact the precision of our findings and/or underestimate the prevalence of the hazards identified. Lastly, the AHS is conducted predominantly in English and Spanish languages (95% of households in the 2019 AHS data). Findings may not be generalizable to the small proportion of U.S. households speaking other languages. In spite of these limitations, a major strength of the HEQI is its accessibility for widespread adoption. The HEQI is based on AHS data that is nationally-representative, publicly-available, and collected biennially by the U.S. Census Bureau. In addition, AHS items in the HEQI are available across survey cycles since 2011 and asked of all occupied households. Therefore, the HEQI can be used in longitudinal analyses to evaluate HEQI trends across U.S. households.

## Conclusions

Our study demonstrated the utility of the HEQI for tracking and evaluating multiple healthy housing domains and identifying vulnerable households at the national level. HEQI findings can contribute to national annual housing reports, such as the Worst Case Housing Needs report to Congress [85] and the Joint Center for Housing Studies’ State of the Nation’s Housing report [74]. In addition, the HEQI expands the portfolio of housing and health disparities research to include environmental health risk factors in the home. Previous studies documenting socioeconomic, racial/ethnic, and nativity-related disparities in housing quality relied on single AHS items or indices that have limited environmental risk factors [7, 86–88]. In addition, national surveys such as the American Community Survey (ACS), U.S. Rental Housing Finance Survey (RHFS), and Residential Energy Consumption Survey (RECS), as well as physical inspection databases like HUD’s Real Estate Assessment Center (REAC) lack key data on environmental risk factors, which then severely limits the ability to identify vulnerable households and inform areas for intervention. The HEQI can be used to inform gaps in these federal data collection programs to capture health-relevant attributes. For example, HEQI findings can inform how the REAC program captures development- or unit-level data for hazard identification or program evaluation. Similarly, the inclusion of HEQI risk factors in the ACS data could facilitate opportunities to better understand how population- and neighborhood-level attributes shape residential environmental exposures.

In conclusion, while we recognize that the addition of residential environmental health questions to existing national surveys may pose financial and administrative challenges, mounting evidence linking residential environmental exposures with adverse health outcomes underscore the need for this data. Furthermore, the most disadvantaged households continue to bear the disproportionate burden of substandard housing conditions, hazardous exposures, and related adverse health outcomes. National environmental health indices that can capture the multiple dimensions of healthy housing enable opportunities to identify vulnerable households and appropriately tailor interventions. Additionally, these indices can be used to benchmark progress over time. Improving housing and indoor environmental quality is a key facet of environmental and climate justice and on the path to advancing health equity.

## Supplementary Information

**Additional file 1: Table S1. **Housing and Environmental Quality Index (HEQI) domains and variable response Items, American Housing Survey 2011-2019 PUF national file. **Tables S2.** Polychoric correlation matrix of domain and item-specific HEQI scores, American Housing Survey 2019 PUF national file (*N*=51,993). Note: Blue cells indicate positive correlations and red cells indicate negative correlations. **Table S3. **Distribution of U.S. households with at least one HEQI risk factor in each domain by unit square footage and normalized leakage (NL) indicator, American Housing Survey 2019 PUF national file (sample *N*=51,993). **Table S4. **Distribution of U.S. households with at least one HEQI risk factor in each domain by status of children (<18 years old) in household, American Housing Survey 2019 PUF national file (sample *N*=51,993). **Appendix 1. **Creating a high building leakage indicator  

## Data Availability

The American Housing Survey 2019 National Public Use Flat (PUF) dataset supporting the conclusions of this article is available in the U.S. Census survey data repository, [https://www.census.gov/programs-surveys/ahs/data.html].

## Abbreviations

AHS: American Housing Survey
HEQI: Housing and Environmental Quality Index
HUD: U.S Department of Housing and Urban Development
PQI: Poor Quality Index
REAC: Real Estate Assessment Center
WHO: World Health Organization
