# Supplementary material for: Development of a multidimensional housing and environmental quality index (HEQI): application to the American Housing Survey
Source: Environ Health. 2022 May 24;21:56. doi: 10.1186/s12940-022-00866-8 (PMC9128206; doi:10.1186/s12940-022-00866-8)
Supplement: Supplementary file 1 — Additional file 1: Table S1. Housing and Environmental Quality Index (HEQI) domains and variable response Items, American Housing Survey 2011-2019 PUF national file. Tables S2. Polychoric correlation matrix of domain and item-specific HEQI scores, American Housing Survey 2019 PUF national file (N=51,993). Note: Blue cells indicate positive correlations and red cells indicate negative correlations. Table S3. Distribution of U.S. households with at least one HEQI risk factor in each domain by unit square footage and normalized leakage (NL) indicator, American Housing Survey 2019 PUF national file (sample N=51,993). Table S4. Distribution of U.S. households with at least one HEQI risk factor in each domain by status of children (<18 years old) in household, American Housing Survey 2019 PUF national file (sample N=51,993). Appendix 1. Creating a high building leakage indicator [file 12940_2022_866_MOESM1_ESM.docx]

**Supplemental Information**

**Development of a Multidimensional Housing and Environmental Quality Index (HEQI):**

**Application to the American Housing Survey**

MyDzung T. Chu^a,b^, Andrew Fenelon^c^, Judith Rodriguez^d,e^, Ami R. Zota^a^*^†^*, Gary Adamkiewicz^d^*^†^*

^a^ Department of Environmental and Occupational Health, Milken Institute School of Public Health, The George Washington University, Washington, DC, USA

^b^ Institute for Clinical Research and Health Policy Studies, Tufts Medical Center, Boston, MA, USA

^c^ School of Public Policy and Department of Sociology and Criminology, Penn State University, University Park, PA, USA

^d^ Department of Architecture, Harvard University Graduate School of Design, Cambridge, MA, USA

^e^ Department of Environmental Health, Harvard T.H. Chan School of Public Health, Boston, MA, USA

*^†^ Dr. Zota and Dr. Adamkiewicz share senior authorship.*

**Table S1.** Housing and Environmental Quality Index (HEQI) Domains and Variable Response Items, American Housing Survey 2011-2019 PUF national file

| **No.** | **Domains** | **American Housing Survey** | | | | |  |
| --- | --- | --- | --- | --- | --- | --- | --- |
|  |  | **2011** | **2013** | **2015** | **2017** | **2019** |  |
| **1** | **Indoor air quality** |  |  |  |  |  |  |
|  | **Household fuel combustion** |  |  |  |  |  |  |
|  | Cooking fuel: Has piped gas or LP gas | CFUEL = 2 | CFUEL = 2 | COOKFUEL = 2-3 | COOKFUEL = 2-3 | COOKFUEL = 2-3 |  |
|  | Heating fuel: Kerosene or other liquid fuel | HFUEL = 4 | HFUEL = 4 | HEATFUEL = 5 | HEATFUEL = 5 | HEATFUEL = 5 |  |
|  | Heating fuel: Coal or coke | HFUEL = 5 | HFUEL = 5 | HEATFUEL = 6 | HEATFUEL = 6 | HEATFUEL = 6 |  |
|  | Heating fuel: Wood | HFUEL = 6 | HFUEL = 6 | HEATFUEL = 7 | HEATFUEL = 7 | HEATFUEL = 7 |  |
|  | Heating type: Cooking stove to heat home | HEQUIP = 14 | HEQUIP = 14 | HEATTYPE = 14 | HEATTYPE = 14 | HEATTYPE = 14 |  |
|  | Heating type: Fireplace without inserts | HEQUIP = 11 | HEQUIP = 11 | HEATTYPE = 11 | HEATTYPE = 11 | HEATTYPE = 11 |  |
|  | Heating type: Has unvented room heaters | HEQUIP = 7 | HEQUIP = 7 | HEATTYPE = 7 | HEATTYPE = 7 | HEATTYPE = 7 |  |
|  | Unit has a useable fireplace | FPLWK = 1 | FPLWK = 1 | FIREPLACE = 1-3 | FIREPLACE = 1-3 | FIREPLACE = 1-3 |  |
| **2** | **Dampness and mold** |  |  |  |  |  |  |
|  | **Mold** (in last 12 months) |  |  |  |  |  |  |
|  | Mold in bathroom | MOLDBATH = 1 | NA | MOLDBATH = 1 | MOLDBATH = 1 | MOLDBATH = 1 |  |
|  | Mold in bedroom | MOLDBEDRM = 1 | NA | MOLDBEDRM = 1 | MOLDBEDRM = 1 | MOLDBEDRM = 1 |  |
|  | Mold in kitchen | MOLDKITCH = 1 | NA | MOLDKITCH = 1 | MOLDKITCH = 1 | MOLDKITCH = 1 |  |
|  | Mold in living room | MOLDLROOM = 1 | NA | MOLDLROOM = 1 | MOLDLROOM = 1 | MOLDLROOM = 1 |  |
|  | Mold in other room | MOLDOTHER = 1 | NA | MOLDOTHER = 1 | MOLDOTHER = 1 | MOLDOTHER = 1 |  |
|  | **Dampness**  (in last 12 months) |  |  |  |  |  |  |
|  | Outside water leak from roof | RLEAK = 1 | RLEAK = 1 | LEAKOROOF = 1 | LEAKOROOF = 1 | LEAKOROOF = 1 |  |
|  | Outside water leak from wall or closed window or door | WLEAK = 1 | WLEAK = 1 | LEAKOWALL = 1 | LEAKOWALL = 1 | LEAKOWALL = 1 |  |
|  | Outside water leak from basement | BLEAK = 1 | BLEAK = 1 | LEAKOBASE = 1 | LEAKOBASE = 1 | LEAKOBASE = 1 |  |
|  | Water leak with unknown inside source | NLEAK2 = 1 | NLEAK2 = 1 | LEAKIDK = 1 | LEAKIDK = 1 | LEAKIDK = 1 |  |
|  | Water leak from broken water heater | WTRHRL = 1 | WTRHRL = 1 | LEAKIWATH = 1 | LEAKIWATH = 1 | LEAKIWATH = 1 |  |
|  | Water leak from somewhere else outside | OTLEAK = 1 | OTLEAK = 1 | LEAKOOTH = 1 | LEAKOOTH = 1 | LEAKOOTH = 1 |  |
|  | Water leak from pipes leaking | PILEAK = 1 | PILEAK = 1 | LEAKIPIPE = 1 | LEAKIPIPE = 1 | LEAKIPIPE = 1 |  |
|  | Water leak from own plumbing fixtures | PLEAK = 1 | PLEAK = 1 | LEAKIPLUM = 1 | LEAKIPLUM = 1 | LEAKIPLUM = 1 |  |
|  | Water leak from somewhere else inside | NLEAK1 = 1 | NLEAK1 = 1 | LEAKIOTH = 1 | LEAKIOTH = 1 | LEAKIOTH = 1 |  |
| **3** | **Pests and allergens** |  |  |  |  |  |  |
|  | Evidence of rats (daily or weekly) | RATFREQ = 1-2 | RATFREQ = 1-2 | RODENT = 1-2 | RODENT = 1-2 | RODENT = 1-2 |  |
|  | Evidence of roaches (daily or weekly) | ROACHFRQ = 1-2 | ROACHFRQ = 1-2 | ROACH = 1-2 | ROACH = 1-2 | ROACH = 1-2 |  |
| **4** | **Lead** |  |  |  |  |  |  |
|  | **Lead paint risk:** Peeling paint larger than 8 x 11 AND home built before 1980 | BIGP = 1 AND BUILT = 1979 or earlier | BIGP = 1 AND BUILT = 1979 or earlier | BIGP = 1 AND BUILT = 1979 or earlier | PAINTPEEL = 1 AND YRBUILT = 1979 or earlier | PAINTPEEL = 1 AND YRBUILT = 1979 or earlier |  |
| **5** | **High indoor temperatures** |  |  |  |  |  |  |
|  | No central AC and No room or window AC unit | AIR = 2 | AIR= 2 | ACPRIMARY = 1-4, 12 | ACPRIMARY = 1-4, 12 | ACPRIMARY = 1-4, 12 |  |
| **6** | **Low indoor temperatures** |  |  |  |  |  |  |
|  | Unit was uncomfortable cold for 24+ hours | FREEZE = 1 | FREEZE = 1 | COLD = 1 | COLD = 1 | COLD = 1 |  |
|  | Main heating equipment broke down 1+ times for 6 hours or more (0-8) | NUMCOLD = 3- 8+ | NUMCOLD = 3- 8+ | COLDEQFREQ = 3- 8+ | COLDEQFREQ = 3- 8+ | COLDEQFREQ = 3- 8+ |  |
| **7** | **Household crowding** |  |  |  |  |  |  |
|  | **Severe Overcrowding:**  Occupancy-to-Room Ratio > 1.5 | PER / ROOMS | PER / ROOMS | NUMPEOPLE / TOTROOMS | NUMPEOPLE / TOTROOMS | NUMPEOPLE / TOTROOMS |  |
| **8** | **Injury hazards** |  |  |  |  |  |  |
|  | **Electrical** |  |  |  |  |  |  |
|  | No electrical wiring | NOWIRE = 3 | NOWIRE = 3 | NOWIRE = 3 | NOWIRE = 3 | NOWIRE = 3 |  |
|  | Electrical wiring exposed | NOWIRE = 2 | NOWIRE = 2 | NOWIRE = 2 | NOWIRE = 2 | NOWIRE = 2 |  |
|  | Not every room has working electrical plug | PLUGS = 2 | PLUGS = 2 | PLUGS = 2 | PLUGS = 2 | PLUGS = 2 |  |
|  | Fuse(s) blown or circuit breakers tripped 2+ times in the last 3 months | NUMBLOW = 3- 8+ | NUMBLOW = 3- 8+ | FUSEBLOW = 3-4 | FUSEBLOW = 3-4 | FUSEBLOW = 3-4 |  |
|  | **Structural Integrity** |  |  |  |  |  |  |
|  | Floor has holes | HOLES = 1 | HOLES = 1 | FLOORHOLE = 1 | FLOORHOLE = 1 | FLOORHOLE = 1 |  |
|  | Walls or ceilings have open holes or cracks wider than dime | CRACKS = 1 | CRACKS = 1 | WALLCRACK = 1 | WALLCRACK = 1 | WALLCRACK = 1 |  |
| **9** | **Inadequate water and sanitation** |  |  |  |  |  |  |
|  | **Poor sanitation** |  |  |  |  |  |  |
|  | 1+ toilet breakdowns within last 3 months that lasted 6 hours or more | NUMTLT = 1-7, 8+ | NUMTLT = 1-7, 8+ | NOTOILFREQ = 1-7, 8+ | NOTOILFREQ = 1-7, 8+ | NOTOILFREQ = 1-7, 8+ |  |
|  | 1+ sewer breakdowns within last 3 months that last 6 hours or more | NUMSEW = 1-7, 8+ | NUMSEW = 1-7, 8+ | SEWBREAK = 1-4+ | SEWBREAK = 1-4+ | SEWBREAK = 1-4+ |  |
|  | Unit has NO bathtub OR shower OR no flush toilet | TOILET = 2 OR BSINK = 2 OR TUB = 2 | TOILET = 2 OR BSINK = 2 OR TUB = 2 | BATHROOMS = 7-13 | BATHROOMS = 7-13 | BATHROOMS = 7-13 |  |
|  | Unit does NOT have working kitchen sink | SINK = 2 | SINK = 2 | KITCHSINK = 2 | KITCHSINK = 2 | KITCHSINK = 2 |  |
|  | **Water quality and quantity** |  |  |  |  |  |  |
|  | Unit has NO hot/cold running water | HOTPIP = 2 | HOTPIP = 2 | HOTWATER = 7 | HOTWATER = 7 | HOTWATER = 7 |  |
|  | Unit completely without running water in the last 90 days | IFDRY = 1 | IFDRY = 1 | NOWAT = 1 | NOWAT = 1 | NOWAT = 1 |  |
|  | Non-public drinking water sources (e.g. individual wells) | WATER = 2-7 | WATER = 2-7 | WATSOURCE = 2-3 | WATSOURCE = 2-3 | WATSOURCE = 2-3 |  |
| **10** | **Ventilation** |  |  |  |  |  |  |
|  | ^High building leakage | UNITSF FLOORS*3 BUILT POOR <=150% Climate Zone estimated at Division-level CELLAR = basement/crawl space OR slab.  Duct location  estimated at the Division-level | UNITSF FLOORS*3 BUILT POOR <=150% Climate Zone estimated at Division-level CELLAR = basement/crawl space OR slab.  Duct location  estimated at the Division-level | UNITSIZE STORIES*3 YRBUILT PERPOVLVL <=150% Climate Zone estimated at Division-level CELLAR = basement/crawl space OR slab.  Duct location  estimated at the Division-level" | UNITSIZE STORIES*3 YRBUILT PERPOVLVL <=150% Climate Zone estimated at Division-level CELLAR = basement/crawl space OR slab.  Duct location  estimated at the Division-level" | UNITSIZE STORIES*3 YRBUILT PERPOVLVL <=150% Climate Zone estimated at Division-level CELLAR = basement/crawl space OR slab.  Duct location  estimated at the Division-level" |  |
|  | Normalized Leakage (NL): 2.6 +: |  |  |  |  |  |  |
| ^ Methods to develop the building leakage indicator was adapted from Chan, W. R., Joh, J., & Sherman, M. H. (2013)  of air leakage measurements of US houses. Energy and Buildings, 66, 616-625. | | | | | | | |

**Tables S2.** Polychoric correlation matrix of domain and item-specific HEQI counts, American Housing Survey 2019 PUF national file (N=51,993)

Note: Blue cells indicate positive correlations and red cells indicate negative correlations.

| **Ten HEQI Domains** | Cumulative total | Household fuel combustion | Dampness and mold | Pests and allergens | High indoor temperatures | Low indoor temperatures | Severe crowding | High building leakage | Lead paint risk | Injury hazards | Inadequate water/sanitation |
| --- | --- | --- | --- | --- | --- | --- | --- | --- | --- | --- | --- |
|  |  |  |  |  |  |  |  |  |  |  |  |
| Cumulative total | 1.00 |  |  |  |  |  |  |  |  |  |  |
| Household fuel combustion | 0.63 | 1.00 |  |  |  |  |  |  |  |  |  |
| Dampness and mold | 0.44 | 0.00 | 1.00 |  |  |  |  |  |  |  |  |
| Pests and allergens | 0.23 | -0.05 | 0.13 | 1.00 |  |  |  |  |  |  |  |
| High indoor temperatures | 0.27 | 0.01 | 0.00 | 0.00 | 1.00 |  |  |  |  |  |  |
| Low indoor temperatures | 0.31 | 0.00 | 0.16 | 0.11 | 0.04 | 1.00 |  |  |  |  |  |
| Severe crowding | 0.07 | -0.01 | 0.01 | 0.05 | 0.02 | 0.01 | 1.00 |  |  |  |  |
| High building leakage | 0.10 | -0.10 | 0.00 | 0.05 | 0.02 | 0.03 | 0.01 | 1.00 |  |  |  |
| Lead paint risk | 0.17 | -0.02 | 0.16 | 0.12 | 0.02 | 0.12 | 0.01 | 0.02 | 1.00 |  |  |
| Injury hazards | 0.38 | -0.01 | 0.17 | 0.14 | 0.02 | 0.13 | 0.02 | 0.03 | 0.17 | 1.00 |  |
| Inadequate water/  sanitation | 0.33 | 0.00 | 0.09 | 0.05 | 0.02 | 0.07 | 0.01 | 0.01 | 0.05 | 0.06 | 1.00 |

| **Household fuel combustion** | Domain total | Cooking fuel: Has piped gas or LP gas | Heat fuel: Kerosene or other liquid fuel | Heat fuel: Coal or coke | Heat fuel: Wood | Heat type: Cooking stove to heat home | Heat type: Fireplace without inserts | Heat type: Unvented room heaters | Useable fireplace |
| --- | --- | --- | --- | --- | --- | --- | --- | --- | --- |
| Domain total | 1.00 |  |  |  |  |  |  |  |  |
| Cooking fuel: Has piped gas or LP gas | 0.74 | 1.00 |  |  |  |  |  |  |  |
| Heat fuel: Kerosene or other liquid fuel | 0.06 | -0.02 | 1.00 |  |  |  |  |  |  |
| Heat fuel: Coal or coke | 0.02 | -0.01 | 0.00 | 1.00 |  |  |  |  |  |
| Heat fuel: Wood | 0.11 | -0.02 | 0.00 | 0.00 | 1.00 |  |  |  |  |
| Heat type: Cooking stove to heat home | 0.03 | 0.00 | 0.00 | 0.00 | 0.01 | 1.00 |  |  |  |
| Heat type: Fireplace without inserts | 0.04 | 0.01 | 0.00 | 0.00 | 0.08 | 0.00 | 1.00 |  |  |
| Heat type: Unvented room heaters | 0.08 | 0.01 | 0.37 | 0.00 | -0.01 | 0.00 | 0.00 | 1.00 |  |
| Useable fireplace | 0.72 | 0.10 | -0.02 | 0.00 | 0.01 | -0.01 | 0.03 | -0.03 | 1.00 |

| **Dampness and mold** | Domain total | Mold in bathroom | Mold in bedroom | Mold in kitchen | Mold in living room | Mold in other room | Outside water leak from roof | Outside water leak from wall or closed window or door | Outside water leak from basement | Water leak with unknown inside source | Water leak from broken water heater | Water leak from somewhere else outside | Water leak from pipes leaking | Water leak from own plumbing fixtures | Water leak from somewhere else inside |
| --- | --- | --- | --- | --- | --- | --- | --- | --- | --- | --- | --- | --- | --- | --- | --- |
| Domain total | 1.00 |  |  |  |  |  |  |  |  |  |  |  |  |  |  |
| Mold in bathroom | 0.30 | 1.00 |  |  |  |  |  |  |  |  |  |  |  |  |  |
| Mold in bedroom | 0.22 | 0.24 | 1.00 |  |  |  |  |  |  |  |  |  |  |  |  |
| Mold in kitchen | 0.18 | 0.27 | 0.26 | 1.00 |  |  |  |  |  |  |  |  |  |  |  |
| Mold in living room | 0.16 | 0.19 | 0.33 | 0.30 | 1.00 |  |  |  |  |  |  |  |  |  |  |
| Mold in other room | 0.17 | 0.09 | 0.08 | 0.08 | 0.06 | 1.00 |  |  |  |  |  |  |  |  |  |
| Outside water leak from roof | 0.49 | 0.09 | 0.07 | 0.06 | 0.06 | 0.05 | 1.00 |  |  |  |  |  |  |  |  |
| Outside water leak from wall or closed window or door | 0.31 | 0.08 | 0.07 | 0.07 | 0.07 | 0.04 | 0.07 | 1.00 |  |  |  |  |  |  |  |
| Outside water leak from basement | 0.36 | 0.01 | 0.01 | 0.00 | 0.00 | 0.01 | 0.03 | 0.04 | 1.00 |  |  |  |  |  |  |
| Water leak with unknown inside source | 0.13 | 0.04 | 0.04 | 0.05 | 0.02 | 0.03 | 0.03 | 0.04 | 0.00 | 1.00 |  |  |  |  |  |
| Water leak from broken water heater | 0.19 | 0.03 | 0.02 | 0.02 | 0.02 | 0.01 | 0.03 | 0.02 | 0.00 | 0.00 | 1.00 |  |  |  |  |
| Water leak from somewhere else outside | 0.26 | 0.06 | 0.06 | 0.03 | 0.06 | 0.05 | 0.01 | 0.02 | 0.00 | 0.03 | 0.01 | 1.00 |  |  |  |
| Water leak from pipes leaking | 0.42 | 0.10 | 0.06 | 0.10 | 0.05 | 0.04 | 0.06 | 0.06 | 0.02 | 0.00 | 0.03 | 0.05 | 1.00 |  |  |
| Water leak from own plumbing fixtures | 0.33 | 0.08 | 0.05 | 0.07 | 0.03 | 0.04 | 0.05 | 0.05 | 0.03 | 0.00 | 0.02 | 0.03 | 0.06 | 1.00 |  |
| Water leak from somewhere else inside | 0.34 | 0.06 | 0.05 | 0.04 | 0.03 | 0.03 | 0.05 | 0.05 | 0.01 | 0.00 | 0.00 | 0.05 | 0.01 | 0.01 | 1.00 |

| **Pests and allergens** | Domain total | Evidence of rats (daily or weekly) | Evidence of roaches (daily or weekly) |
| --- | --- | --- | --- |
| Domain total | 1.00 |  |  |
| Evidence of rats (daily or weekly) | 0.60 | 1.00 |  |
| Evidence of roaches (daily or weekly) | 0.86 | 0.18 | 1.00 |

| **Low indoor temperatures** | Domain total | Unit was uncomfortable cold for 24+ hours | Main heating equipment broke down 1+ times for 6 hours or more (0-8) |
| --- | --- | --- | --- |
| Domain total | 1.00 |  |  |
| Unit was uncomfortable cold for 24+ hours | 1.00 | 1.00 |  |
| Main heating equipment broke down 1+ times for 6 hours or more (0-8) | 0.63 | 0.60 | 1.00 |

| **Injury hazards** | Domain total | No electrical wiring | Electrical wiring exposed | Not every room has working electrical plug | Fuse(s) blown or circuit breakers tripped 2+ times in the last 3 mo. | Floor has holes | Walls or ceilings have open holes or cracks wider than dime |
| --- | --- | --- | --- | --- | --- | --- | --- |
| Domain total | 1.00 |  |  |  |  |  |  |
| No electrical wiring | 0.06 | 1.00 |  |  |  |  |  |
| Electrical wiring exposed | 0.47 | 0.00 | 1.00 |  |  |  |  |
| Not every room has working electrical plug | 0.41 | 0.13 | 0.24 | 1.00 |  |  |  |
| Fuse(s) blown or circuit breakers tripped 2+ times in the last 3 mo. | 0.51 | 0.00 | 0.02 | 0.04 | 1.00 |  |  |
| Floor has holes | 0.30 | 0.00 | 0.04 | 0.05 | 0.06 | 1.00 |  |
| Walls or ceilings have open holes or cracks wider than dime | 0.64 | 0.00 | 0.05 | 0.06 | 0.09 | 0.24 | 1.00 |

| **Water and Sanitation** | Domain total | 1+ toilet breakdowns within last 3 months that lasted 6 hours or more | 1+ sewer breakdowns within last 3 months that last 6 hours or more | Unit has NO bathtub OR shower OR no flush toilet | Unit does NOT have working kitchen sink | Unit has NO hot/cold running water | Unit completely without running water in last 90 days | Source of water for unit not from Public or Private system |
| --- | --- | --- | --- | --- | --- | --- | --- | --- |
| Domain total | 1.00 |  |  |  |  |  |  |  |
| 1+ toilet breakdowns within last 3 months that lasted 6 hours or more | 0.32 | 1.00 |  |  |  |  |  |  |
| 1+ sewer breakdowns within last 3 months that last 6 hours or more | 0.28 | 0.20 | 1.00 |  |  |  |  |  |
| Unit has NO bathtub OR shower OR no flush toilet | 0.09 | 0.00 | 0.02 | 1.00 |  |  |  |  |
| Unit does NOT have working kitchen sink | 0.12 | 0.00 | 0.00 | 0.16 | 1.00 |  |  |  |
| Unit has NO hot/cold running water | 0.14 | 0.02 | 0.02 | 0.17 | 0.08 | 1.00 |  |  |
| Unit completely without running water in last 90 days | 0.46 | 0.13 | 0.07 | 0.02 | 0.00 | -0.01 | 1.00 |  |
| Source of water for unit not from Public or Private system | 0.78 | -0.01 | 0.00 | 0.01 | -0.01 | 0.01 | 0.03 | 1.00 |

**Table S3.** Distribution of U.S. households with at least one HEQI risk factor in each domain by unit square footage and normalized leakage (NL) indicator, American Housing Survey 2019 national public file (sample N=51,993)

|  | **All Households** | **<1,000 Ft^2^** | | **1,001-1,500 Ft^2^** | | **1,501+ Ft^2^** | |
| --- | --- | --- | --- | --- | --- | --- | --- |
|  |  | **NL < 1.0** | **NL ≥ 1** | **NL < 1.0** | **NL ≥ 1** | **NL < 1.0** | **NL ≥ 1** |
| Weighted N (%) | (N=117,284,000) | (N=5,889,007) | (N=21,442,847) | (N=11,825,730) | (N=20,810,484) | (N=37,322,791) | (N=19,992,930) |
| Cumulative | 92,043,000 (78.5%) | 3,433,000 (58.3%) | 15,441,000 (72.0%) | 8,090,000 (68.4%) | 16,065,000 (77.2%) | 31,956,000 (85.6%) | 17,058,000 (85.3%) |
| Household fuel combustion | 71,962,000 (61.4%) | 2,054,000 (34.9%) | 9,069,000 (42.3%) | 6,110,000 (51.7%) | 11,936,000 (57.4%) | 28,704,000 (76.9%) | 14,087,000 (70.5%) |
| Dampness and mold | 18,699,000 (15.9%) | 708,000 (12.0%) | 3,711,000 (17.3%) | 1,506,000 (12.7%) | 3,853,000 (18.5%) | 4,779,000 (12.8%) | 4,141,000 (20.7%) |
| Pests and allergens | 4,949,000 (4.2%) | 242,000 (4.1%) | 1,780,000 (8.3%) | 315,000 (2.7%) | 1,160,000 (5.6%) | 752,000 (2%) | 7,01,000 (3.5%) |
| Lead paint risk | 1,952,000 (1.7%) | 9,000 (0.2%) | 687,000 (3.2%) | 59,000 (0.5%) | 528,000 (2.5%) | 159,000 (0.4%) | 509,000 (2.5%) |
| High indoor temperature | 10,344,000 (8.8%) | 748,000 (12.7%) | 3,023,000 (14.1%) | 739,000 (6.3%) | 2,212,000 (10.6%) | 1,933,000 (5.2%) | 1,687,000 (8.4%) |
| Low indoor temperature | 6,841,000 (5.8%) | 279,000 (4.7%) | 1,635,000 (7.6%) | 479,000 (4%) | 1,606,000 (7.7%) | 1,496,000 (4%) | 1,347,000 (6.7%) |
| Severe overcrowding | 507,000 (0.4%) | 55,000 (0.9%) | 259,000 (1.2%) | 13,000 (0.1%) | 113,000 (0.5%) | 39,000 (0.1%) | 28,000 (0.1%) |
| Injury hazards | 13,959,000 (11.9%) | 620,000 (10.5%) | 3,275,000 (15.3%) | 1,064,000 (9%) | 2,928,000 (14.1%) | 3,597,000 (9.6%) | 2,476,000 (12.4%) |
| Inadequate water and sanitation | 16,802,000 (14.3%) | 601,000 (10.2%) | 2,406,000 (11.2%) | 1,494,000 (12.6%) | 2,716,000 (13.1%) | 5,834,000 (15.6%) | 3,751,000 (18.8%) |

Footnote: NL <1.0 indicate building airtightness. Estimates are weighted to the U.S. household population and rounded to the nearest thousandth.

For unit square footage, we took the midpoint of the reported unit size range, with values capped at 250 feet^2^ and 4,500 feet^2^. For households missing unit size, we multiplied the reported number of rooms by the median room size (19.35 m^2^) for households that had available unit size data.

Abbreviation: NL = Normalized leakage.

**Table S4.** Distribution of U.S. households with at least one HEQI risk factor in each domain by status of children (<18 years old) in household, American Housing Survey 2019 national public file (sample N=51,993)

|  | **All Households** | **Households without Children** | **Households with Children** |
| --- | --- | --- | --- |
| Weighted N (%) | (N=117,284,000) | (N=83,142,397) | (N=34,141,394) |
| Cumulative | 92,043,000 (78.5%) | 64,189,000 (77.2%) | 27,854,000 (81.6%) |
| Household fuel combustion | 71,962,000 (61.4%) | 49,636,000 (59.7%) | 22,326,000 (65.4%) |
| Dampness and mold | 18,699,000 (15.9%) | 12,218,000 (14.7%) | 6,481,000 (19%) |
| Pests and allergens | 4,949,000 (4.2%) | 3,053,000 (3.7%) | 1,895,000 (5.6%) |
| Lead paint risk | 1,952,000 (1.7%) | 1,333,000 (1.6%) | 619,000 (1.8%) |
| High indoor temperature | 10,344,000 (8.8%) | 7,874,000 (9.5) | 2,470,000 (7.2) |
| Low indoor temperature | 6,841,000 (5.8%) | 4,670,000 (5.6%) | 2,171,000 (6.4%) |
| Severe overcrowding | 507,000 (0.4%) | 72,000 (0.1%) | 434,000 (1.3%) |
| Injury hazards | 13,959,000 (11.9%) | 9,223,000 (11.1%) | 4,736,000 (13.9%) |
| Inadequate water and sanitation | 16,802,000 (14.3%) | 12,194,000 (14.7%) | 4,608,000 (13.5%) |
| High building leakage | 2,813,000 (2.4%) | 2,361,000 (2.8%) | 452,000 (1.3%) |

Footnote: Estimates are weighted to the U.S. household population and rounded to the nearest thousandth.

**Appendix 1. *Creating a High Building Leakage Indicator***

Poor building airtightness has been associated with a higher risk for water leakage, dampness and mold, pest problems, and infiltration from other units [1]. This is measured using the Normalized Leakage (NL) indicator, a measure of tightness of the building envelope relative to its size and height [2]. We estimated NL based on methods by Chan et al. (2013) using the Lawrence Berkeley National Laboratory’s Residential Diagnostic Database (ResDB) national dataset of air leakage measurements from 134,000 single-family detached homes [3]. They found that housing information on year built, climate zones, eligibility for weatherization assistance programs, energy efficiency rating, housing dimensions (i.e. floor area, house height), foundation type, and duct location were important predictors of NL. To estimate the NL distribution in the national public AHS data, we extracted these parameters from the AHS, coded them to be consistent with the data format in the Chan et al. (2013) paper whenever possible, and applied the NL model coefficients (Table 1). Year built was kept as 10-year increments (<1960, 1960-1969, 1970-1979, 1980-1989, 1990-1999, and 2000 and after). For floor area (m^2^), we took the midpoint of the reported unit size range, with values capped at 250 feet^2^ and 4,500 feet^2^, and converted values to meter^2^. For the 27% households missing unit size, we multiplied the reported number of rooms by the median room size (19.35 m^2^) for households that had available unit size data. For house height, we multiplied the number of stories by 3 meters, the estimated height for each floor that has been cited in the literature [3, 4]. The number of stories was capped at seven. For foundation type, the AHS did not have information whether crawlspaces were vented or conditioned. We assumed all crawlspaces were unconditioned and vented, which is more prevalent in US households than unvented and conditioned basements [3].

Also, the AHS data does not collect information about WAP eligibility, energy efficiency ratings, climate zones, or duct type, and thus we made several assumptions and relied on external data sources, consistent with previous approaches [3, 5].

- For WAP eligibility, we assumed homes within a federal poverty level of 150% were eligible.
- The influence of energy efficiency was assumed to be negligible.
- Data on climate zone designations (1-7) by moisture regime (A-C, AK) came from the International Energy Conservation Code (IECC) for the eight U.S. climate regions by county [6]. Since the smallest geography in the public AHS data is U.S. divisions (i.e. East North Central, Middle Atlantic, Mountain-Pacific, New England, South Atlantic/East South Central, West North Central, and West South Central), we took the median of the county-level climate zone values for each divisions and then applied the NL model coefficient for each zone designation. For divisions with multiple zone designations (e.g. Mountain-Pacific division had four designations: 1 A, 7AK, 5 B, and 4 C), we used data on household size from the 2010 Decennial Census to weight the coefficient estimate for each zone within each division, with the sum of weights equaling to 1 (Table A1).
- Data on duct type came from home inspection and energy audit data from the Home Energy Score [7] that approximated the fraction of housing units with ducts in the conditioned space, unconditioned attic or basement, or in a crawlspace (assumed to be vented) for each state [3]. We estimated fractions for each US divisions by using the number of states in each division as the weights, with the sum of weights equaling to 1 (Table A2).

We applied the NL leakage model from Chan et al. (2013) to our AHS data as follows [3]. Coefficients can be found in Table 1.

ln(NL)_(i)_ = ß_area_FloorArea_(i)_ + ß_h_Height + ß_year_Year Indicator_(i)_ +ß_wap_WAP_(i)_ + ß_cz_ClimateZone Indicator_(d)_ + ß_slab_Slab_(i)_ + ß_floor2_ßFloor2_(i)_ + ß_cond_Cond_(d)_ + ß_duct1_Duct1_(d)_ + ß_duct2_Duct2_(d)_ + *e*

= [-2.08E-03*FloorArea_(i)_] + [6.38E-02*Height_(i)_] + [-2.50E-01*YrBuilt<1960_(i)_] +

[-4.33E-01* YrBuilt1960-1969_(i)_] + [-4.52E-01* YrBuilt1970-1979_(i)_] + [-6.54E-01* YrBuilt1980-1989_(i)_] + [-9.15E-01* YrBuilt1990-1999_(i)_] + [-1.06E+00* YrBuilt2000+_(i)_] + [4.20E-01*WAP_(i)_] + ß_cz_ClimateZone Indicator_(d)_ + [-0.037*Slab_(i)_] + [0.180*Floor2_(i)_] + ß_cond_Cond_(d)_ + ß_duct1_Duct1_(d)_ + ß_duct2_Duct2_(d)_ + *e*

where *i* represents AHS household, *d* represents US divisions, and *e* is the residual term that is roughly normal distributed N($\mu$ = 0, $\sigma$^2^= 0.2)

For the HEQI, we created an binary indicator of high building leakage consistent with the BC Housing Organization’s guidance [1]: Low leakage: 0-2.50 and High leakage: 2.51+.

**Table A1.** Approximation of Chan et al. 2013 model coefficients for IECC moisture regime and climate zone class for each US Division

| **US Division** | **IECC Moisture Regime (MR)** | **IECC Climate Zone (CZ)** | **Beta^** | **SE^** | **No. Counties per MR-CZ and Division** | **No. Households (HH) per MR-CZ and Division** | **Total HH per Division** | **Weight: Proportion of MR-CZ HH per Division** | **Estimated Beta (weighted)*** | **Estimated SE (weighted)*** |
| --- | --- | --- | --- | --- | --- | --- | --- | --- | --- | --- |
| East North Central | A | 5 | 0.112 | 0.006 | 412 | 43,476 | 43,476 | 1.000 | 0.112 | 0.006 |
| Middle Atlantic | A | 5 | 0.112 | 0.006 | 150 | 103,673 | 103,673 | 1.000 | 0.112 | 0.006 |
| Mountain Pacific | A | 1 | 0.473 | 0.010 | 5 | 91,068 | 275,357 | 0.331 | 0.156 | 0.003 |
|  | AK | 7 | 0.026 | 0.006 | 28 | 9,154 | 275,357 | 0.033 | 0.001 | 0.000 |
|  | B | 5 | -0.009 | 0.007 | 346 | 53,644 | 275,357 | 0.195 | -0.002 | 0.001 |
|  | C | 4 | 0.258 | 0.011 | 52 | 121,491 | 275,357 | 0.441 | 0.114 | 0.005 |
| New England | A | 6 | 0.000 | 0.000 | 66 | 85,355 | 85,355 | 1.000 | 0.000 | 0.000 |
| South Atlantic & East South Central | A | 3 | 0.253 | 0.007 | 1031 | 29,431 | 29,431 | 1.000 | 0.253 | 0.007 |
| West North Central | A | 5 | 0.112 | 0.006 | 557 | 13,615 | 13,615 | 1.000 | 0.112 | 0.006 |
| West South Central | A | 3 | 0.253 | 0.007 | 355 | 34,335 | 43,638 | 0.787 | 0.199 | 0.005 |
|  | B | 3 | -0.038 | 0.008 | 115 | 9,302 | 43,638 | 0.213 | -0.008 | 0.002 |

^Betas and standard errors from Chan et al. 2013 model for each IECC Moisture Regime and Climate Zone [3].

* Weighted estimated betas and standard error for our model.

**Table A2.** Approximation of Chan et al. 2013 model coefficients for duct type for each US Division

| **U.S. Census Divisions** | **RECS 2009 area** | **Proportions per REC area** | | | **Number of states** | **Weights by No. of State per US Division** | | | **Estimated Beta (weighted)*** | | | **Estimated SE (weighted)*** | | |
| --- | --- | --- | --- | --- | --- | --- | --- | --- | --- | --- | --- | --- | --- | --- |
|  |  | Con-ditioned | Uncon-ditioned | Crawl space |  | Con-ditioned | Uncon-ditioned | Crawl space | Con-ditioned | Uncon-ditioned | Crawl space | Con-ditioned | Uncon-ditioned | Crawl space |
| New England | 1. Connecticut, Maine, New Hampshire, Rhode Island, Vermont | 0.14 | 0.83 | 0.02 | 4 | 0.134 | 0.842 | 0.016 | -0.017 | 0.060 | 0.003 | 0.003 | 0.029 | 0.001 |
|  | 2. Massachusetts | 0.11 | 0.89 | 0 | 1 |  |  |  |  |  |  |  |  |  |
| Mid-Atlantic | 3. New York | 0.16 | 0.81 | 0.03 | 1 | 0.177 | 0.790 | 0.030 | -0.022 | 0.056 | 0.005 | 0.005 | 0.027 | 0.001 |
|  | 4. New Jersey | 0.17 | 0.79 | 0.04 | 1 |  |  |  |  |  |  |  |  |  |
|  | 5. Pennsylvania | 0.2 | 0.77 | 0.02 | 1 |  |  |  |  |  |  |  |  |  |
| East North Central | 6. Illinois | 0.7 | 0.26 | 0.05 | 1 | 0.736 | 0.212 | 0.050 | -0.091 | 0.015 | 0.009 | 0.019 | 0.007 | 0.002 |
|  | 7. Indiana, Ohio | 0.72 | 0.23 | 0.04 | 2 |  |  |  |  |  |  |  |  |  |
|  | 8. Michigan | 0.77 | 0.15 | 0.08 | 1 |  |  |  |  |  |  |  |  |  |
|  | 9. Wisconsin | 0.77 | 0.19 | 0.04 | 1 |  |  |  |  |  |  |  |  |  |
| West North Central | 10. Iowa, Minnesota, North Dakota, South Dakota | 0.87 | 0.11 | 0.02 | 4 | 0.813 | 0.159 | 0.029 | -0.101 | 0.011 | 0.005 | 0.021 | 0.005 | 0.001 |
|  | 11. Kansas, Nebraska | 0.71 | 0.25 | 0.04 | 2 |  |  |  |  |  |  |  |  |  |
|  | 12. Missouri | 0.79 | 0.17 | 0.04 | 1 |  |  |  |  |  |  |  |  |  |
| South Atlantic & East South Central | 13. Virginia | 0.53 | 0.41 | 0.06 | 1 | 3.885 | 6.225 | 1.987 | -0.482 | 0.442 | 0.360 | 0.099 | 0.211 | 0.076 |
|  | 14. Delaware, District of Columbia, Maryland, West Virginia | 0.68 | 0.25 | 0.07 | 4 |  |  |  |  |  |  |  |  |  |
|  | 15. Georgia | 0.14 | 0.74 | 0.12 | 1 |  |  |  |  |  |  |  |  |  |
|  | 16. North Carolina, South Carolina | 0.09 | 0.74 | 0.18 | 2 |  |  |  |  |  |  |  |  |  |
|  | 17. Florida | 0.04 | 0.91 | 0.05 | 1 |  |  |  |  |  |  |  |  |  |
|  | 18. Alabama, Kentucky, Mississippi | 0.09 | 0.55 | 0.36 | 3 |  |  |  |  |  |  |  |  |  |
|  | 19. Tennessee | 0.07 | 0.46 | 0.48 | 1 |  |  |  |  |  |  |  |  |  |
| West South Central | 20. Arkansas, Louisiana, Oklahoma | 0.05 | 0.88 | 0.07 | 3 | 0.163 | 2.875 | 0.213 | -0.020 | 0.204 | 0.038 | 0.004 | 0.097 | 0.008 |
|  | 21. Texas | 0.05 | 0.94 | 0.01 | 1 |  |  |  |  |  |  |  |  |  |
| Mountain Pacific | 22. Colorado | 0.51 | 0.43 | 0.06 | 1 | 0.239 | 0.532 | 0.228 | -0.030 | 0.038 | 0.041 | 0.006 | 0.018 | 0.009 |
|  | 23. Idaho, Montana, Utah, Wyoming | 0.48 | 0.42 | 0.1 | 4 |  |  |  |  |  |  |  |  |  |
|  | 24. Arizona | 0.08 | 0.89 | 0.04 | 1 |  |  |  |  |  |  |  |  |  |
|  | 25. Nevada, New Mexico | 0.01 | 0.88 | 0.11 | 2 |  |  |  |  |  |  |  |  |  |
|  | 26. California | 0.02 | 0.88 | 0.09 | 1 |  |  |  |  |  |  |  |  |  |
|  | 27. Alaska, Hawaii, Oregon, Washington | 0.14 | 0.32 | 0.54 | 4 |  |  |  |  |  |  |  |  |  |

* Weighted estimated betas and standard error for our model

**References:**

1. British Columbia Housing, BC Hydro, and City of Vancouver. Illustrated Guide to Achieving Airtightness (2017). Access at: [www.bchousing.org/publications/Illustrated-Guide-Achieving-Airtightness.pdf](http://www.bchousing.org/publications/Illustrated-Guide-Achieving-Airtightness.pdf) (11/01/2021)
2. Sherman, M. H., & McWilliams, J. A. (2007). Air leakage of US homes: Model prediction (No. LBNL-62078). Ernest Orlando Lawrence Berkeley National Laboratory, Berkeley, CA (US). Access at: <https://web.ornl.gov/sci/buildings/conf-archive/2007%20B10%20papers/152_Sherman.pdf>
3. Chan, W. R., Joh, J., & Sherman, M. H. (2013). Analysis of air leakage measurements of US houses. Energy and Buildings, 66, 616-625.
4. Chahine T, Schultz B, Zartarian V, Subramanian SV, Spengler J, Hammitt J, Levy JI. Modeling geographic and demographic variability in residential concentrations of environmental tobacco smoke using national data sets. Journal of Exposure Science & Environmental Epidemiology. 2011 Nov;21(6):646-55.
5. Chan WR, Nazaroff WW, Price PN, Sohn MD, Gadgil AJ. Analyzing a database of residential air leakage in the United States. Atmospheric Environment. 2005 Jun 1;39(19):3445-55.
6. Baechler M, Williamson J, Gilbride T, Cole P, Hefty M, Love PM. Guide to determining climate regions by county (no. PNNL-17211). Richland, WA: Pacific Northwest National Laboratory. 2010. Access at: <https://www.energy.gov/sites/prod/files/2015/10/f27/ba_climate_region_guide_7.3.pdf>. Data access at: <https://www.arcgis.com/home/item.html?id=8e5c3c6e1fa94e379553e199dcc4e777> [downloaded July 2021].
7. United States Department of Energy (2012). “About the home energy score”. Access at: https://betterbuildingssolutioncenter.energy.gov/home-energy-score/home-energy-score-about-score.
